# Vitamin D and VDR Gene Polymorphisms’ Association with Rheumatoid Arthritis in Lithuanian Population

**DOI:** 10.3390/medicina57040346

**Published:** 2021-04-03

**Authors:** Egle Punceviciene, Justina Gaizevska, Rasa Sabaliauskaite, Lina Venceviciene, Alina Puriene, Dalius Vitkus, Sonata Jarmalaite, Irena Butrimiene

**Affiliations:** 1 Clinic of Rheumatology, Traumatology Orthopaedics and Reconstructive Surgery, Institute of Clinical Medicine of the Faculty of Medicine, Vilnius University, M. K. Čiurlionio str. 21, 03101Vilnius, Lithuania; irena.butrimiene@santa.lt; 2 State Research Institute Centre for Innovative Medicine, Santariškių str. 5, 08406 Vilnius, Lithuania; 3 Centre of Rheumatology, Vilnius University Hospital Santaros klinikos, Santariškių str. 2, 08661 Vilnius, Lithuania; 4 Life Sciences Center, Institute of Biosciences, Vilnius University, Saulėtekio av. 7, 10257 Vilnius, Lithuania; justina.gaizevska@nvi.lt (J.G.); sonata.jarmalaite@nvi.lt (S.J.); 5 National Cancer Institute, Santariškių str. 1, 08660 Vilnius, Lithuania; rasa.sabaliauskaite@nvi.lt; 6 Centre of Family Medicine, Vilnius University Hospital Santaros Klinikos, Santariškių str. 2, 08406 Vilnius, Lithuania; lina.venceviciene@santa.lt; 7 Clinic of Internal Medicine, Family Medicine and Oncology, Faculty of Medicine, Vilnius University, M. K. Čiurlionio str. 21, 03101 Vilnius, Lithuania; 8 Institute of Odontology, Faculty of Medicine, Vilnius University, M. K. Čiurlionio str. 21, 03101 Vilnius, Lithuania; alina.puriene@gmail.com; 9 Vilnius University Hospital Žalgiris Clinic, Žalgirio str. 115, 08217 Vilnius, Lithuania; 10 Institute of Biomedical Sciences of the Faculty of Medicine, Vilnius University, M. K. Čiurlionio str. 21, 03101 Vilnius, Lithuania; dalius.vitkus@santa.lt; 11 Centre of Laboratory Medicine, Vilnius University Hospital Santarosklinikos, Santariškių str. 2, 08661 Vilnius, Lithuania

**Keywords:** rheumatoid arthritis, single nucleotide polymorphisms, vitamin D, vitamin D receptor

## Abstract

*Background and Objectives:* Rheumatoid arthritis (RA) is a chronic, inflammatory, autoimmune, multi-factorial disease, in which environmental and genetic factors play a major role. RA is possibly linked to vitamin D deficiency and vitamin D receptor (*VDR*) gene polymorphisms, and research demonstrates that *FokI* variant susceptibility is associated with increased disease risk among Caucasians. The aim of this study was to evaluate vitamin D deficiency prevalence and its correlation to RA clinical parameters, and to determine the possible association of *VDR* gene polymorphisms and RA susceptibility in the Lithuanian population. *Materials and Methods*: Overall, 206 RA patients and 180 age- and sex-matched healthy controls were enrolled at Vilnius University Hospital Santaros Klinikos after informed consent was obtained. The disease activity score 28 C-reactive protein (DAS28 CRP), rheumatoid arthritis impact of disease (RAID) score, and health assessment questionnaire (HAQ) were recorded in RA patients, and 25(OH)D serum levels were evaluated by chemiluminescent microparticle immunoassay for all subjects. Four *VDR* gene polymorphisms, *BsmI*, *FokI*, *ApaI*, and *TaqI*, were assessed using real-time PCR instruments and genotyping assays in both groups. *Results*: The study registered a high prevalence of 25(OH)D deficiency (<50 nmol/L) in RA patients (61.55% (*n* = 127)). The mean serum concentration in RA patients (44.96 ± 21.92 (nmol/L)) was significantly lower than in the healthy controls (54.90 ± 22.82 (nmol/L)), *p* < 0.0001. A significant inverse correlation between vitamin D level, DAS28 CRP, and HAQ scores was confirmed in RA patients, with p < 0.05. Still, there was no significant association between the overall risk of RA disease for any allele or genotype of the four *VDR* loci tested. *Conclusions*: The study confirmed that vitamin D deficiency is prevalent among RA patients and the 25(OH)D level is significantly lower compared with healthy controls. Lower vitamin D concentration was related with increased disease activity and disability scores. However, genetic analysis of four *VDR* polymorphisms did not confer the susceptibility to RA in Lithuanian population.

## 1. Introduction

Rheumatoid arthritis (RA) is a chronic autoimmune inflammatory disease that mostly affects synovial tissue, less commonly occurs as extra-articular manifestations, and leads to higher mortality and disability rates [1]. While the etiology of RA has not yet been fully elucidated, a combination of genetic, epigenetic, and environmental factors are consideredas potential risk factors of the disease that release subsequent changes in the innate and adaptive immune system and the development of RA [2].

Special attention on vitamin D in autoimmune diseases including RA was brought to light during the past decades, explored in in vitro studies, and discussed in scientific literature, revealing its non-classical effects beyond bone metabolism, i.e., calcium homeostasis [3]. Vitamin D is a fat-soluble vitamin, secosteroid, and is mainly synthesized in the human skin from the precursor molecule 7-dehydrocholesterol during UV exposure, although it is also obtained from dietary sources [1]. The active form of vitamin D, 1,25 dihydroxyvitamin D (1,25(OH)2D3), contributes to the regulation and differentiation of the immune cells and leads to potential anti-inflammatory, immunomodulatory, antiproliferative, and even, gene expression-modulating properties [3,4,5]. Vitamin D is involved in the mechanisms of regulation of the immune system, it regulates the action of suppressor T lymphocytes and the synthesis of cytokines, and acts by modulating the processes of cellular apoptosis [6]. In addition, multiple actions on dendritic, T/B cells lead to the decrease in the production of numerous cytokines, i.e., TNF-α, IL-17, IL-6, and IL-1, and the inhibition of IL-2 and interferon γ, which are crucial in etiopathogenesis of RA [7]. These insights clarify why vitamin D deficiency is possibly linked to RA and is associated with higher disease activity and a worse quality of life [8,9].Vitamin D mediates its function by binding to the vitamin D receptor (VDR), which is encoded by the *VDR* gene—a member of nuclear hormone receptors superfamily detected in various immune cell types that is also present on chondrocytes and synovial cells in RA affected joints [5,10].

*VDR* gene single nucleotide polymorphisms (SNP) are one of the most studied gene variants in the background of RA. This gene is located on chromosome 12 (locus geni 12q13.11) and consists of 11 exons, 3 of which form a non-coding 5′ region, and the other8 are responsible for the VDR product. The *VDR* gene has a large promoter region able to generate multiple tissue-specific transcripts [11,12]. To date, there are over 470 SNPs identified in the human *VDR* gene, but the most extensively studied are *TaqI*, *FokI*, *BsmI*, and *ApaI* [12,13]. *BsmI* and *ApaI* are found in intron 8 and *TaqI* is found in exon 9, implying that all three SNPs are located at the 3′-end of the gene and are proved to be in strong linkage disequilibrium. Meanwhile, *FokI* is a start codon polymorphism and may have an indirect effect on 25(OH)D levels [12,14,15]. Data shows that *VDR* gene variants have a complex effect on VDR expression and function itself. In addition, over 11,000 presumptive VDR target genes are involved in miscellaneous physiological roles, like cell proliferation, differentiation, metabolism, and others, enhancing their involvement in a number of diverse diseases and encouraging studies in the etiopathogenesis of RA [15]. RA susceptibility to *VDR* gene polymorphisms differs by geographical latitude and ethnic subgroups, revealing that the *FokI* variant potentially increases disease risk among Caucasians, while BsmI is linked to RA-associated bone loss [12,14].

The aim of this study was to evaluate the prevalence of vitamin D deficiency, its correlation to RA disease activity, and correlation to disability scores, as well as to determine a possible association between *VDR* gene polymorphisms and RA susceptibility in the Lithuanian population.

## 2. Materials and Methods

### 2.1. Subjects

For this case-control study, 206 RA patients (184 females/22 males, mean age 55.01 ± 11.08) and 180 age- and sex-matched healthy controls (163 females/17 males, mean age 53.15 ± 10.68) of the Lithuanian population were enrolled at Vilnius University Hospital Santaros Klinikos (VUHSK) Rheumatology Center after informed consent was obtained according to the permission of Vilnius Regional Biomedical Research Ethics Committee. For autoimmune arthritis subjects, RA diagnosis was established by the American College of Rheumatology/European League Against Rheumatism (ACR/EULAR) 2010 rheumatoid arthritis classification criteria at least 1 year prior to the study inclusion, or by the 1987 ACR classification criteria if diagnosed before the year 2010 [16,17]. Demographic, anthropometric, and clinical/medical data from all RA patients were collected. All RA patients were asked to fill out two provided questionnaires: the health assessment questionnaire (HAQ) and rheumatoid arthritis impact of disease (RAID) score [18,19]. The disease activity score28 C-reactive protein(DAS28 CRP) was measured by counting 28 tender and swollen joints, the visual analogue scale (VAS 100 mm), and the C-reactive protein concentration (CRP, mg/L), and then classified as follows: high disease activity (≥5.1), moderate disease activity (>3.2 to 5.1), low disease activity (2.6 to ≤3.2) and remission (<2.6) [20]. The control group was invited to participate in the VUHSK Family Medicine Center by a family doctor, and if eligible, they were further referred to the Rheumatology Center for the informed consent form signing and data (demographic, anthropometric, and blood samples) collection. All recruited subjects were >18 years old. Subjects with diagnosed cancer (<5 years), other autoimmune comorbidities (e.g., diabetes mellitus, etc.), and pregnancy were excluded. Biochemical tests and genetic analysis blood samples from all subjects were collected, coded, and labeled as required.

### 2.2. Vitamin D Evaluation

All subjects (RA patients and healthy controls) were enrolled from late October until the middle of May, according to known seasonal 25(OH)D serum level variations and their possible influence on disease activity and severity scores. Blood samples were collected using BD Vacutainer Serum Separator Tubes (5 mL) (BD Biosciences, NJ, USA) and prepared by standard procedures. The vitamin D serum level evaluation was performed in VUHSK, Center of Laboratory Medicine using chemiluminescent microplate immunoassay (Architect ci8200, Abbott Laboratories, IL, USA), with the ability to detect 25(OH)D_3_ from 98.6% to 101.1% and 25(OH)D_2_ from 80.5% to 84.4%. Vitamin D concentration was classified as normal (≥75 nmol/L), insufficient (≥50–75 nmol/L), or deficient (<50 nmol/L) [21].

### 2.3. Cell Preparation and DNA Isolation

Blood samples for DNA analysis from all subjects were collected using BD Vacutainer^®^ CPT™mononuclear cell preparation tube sodium citrate vacutainers (8 mL) (BD Biosciences). Peripheral blood mononuclear cells (PBMC) were prepared using the manufacturer’s recommendations and standardized procedures: tubes with blood samples were centrifuged (Centrifuge, Heraeus Megafuge 8 Centrifuge, Thermo Scientific (TS) part of Thermo Fisher Scientific (TFS), Wilmington, DE, USA) by stages, and cell washing steps using phosphate-buffered saline (PBS) (Biochrom, Berlin, Germany) were performed as suggested. Samples with prepared PBMC were stored at −70 °C. For further genetic analysis, DNA isolation was performed using commercial GeneJET Genomic DNA Purification Kit (TFS) by the manufacturer‘s recommendations. Isolated DNA concentration and purity were measured by a 260/280 and 260/230 ratio using a NanoDrop^®^ 2000 spectrophotometer (TS, TFS). Each subject DNA sample was placed into LoBind microcentrifuge 1.5 mL tubes (Eppendorf, Hamburg, Germany), labeled, and stored at −20 °C.

### 2.4. Quantitative Polymerase Chain Reaction (qPCR) and VDR Genotyping

The TaqMan probe-based qPCR analysis was used for sensitive and specific identification of four target *VDR* gene polymorphisms (*TaqI* (rs731236), *BsmI* (rs1544410), *ApaI* (rs7975232), and *FokI* (rs2228570)). This method exploits the 5′ → 3′ nuclease activity of the Taq polymerase to detect and quantify specific PCR products. One pair of unlabeled primers was used to amplify the desired DNA region containing the SNP. In addition, two TaqMan hydrolysis probes (with a different reporter dye and a common quencher dye on the 5′ and 3′ ends, respectively) which differed only at the polymorphic site were designed. One probe was made complementary to the wild-type allele, while the other probe was complementary to the alternative allele. The probes bind to the polymorphic site of interest during the annealing phase of the PCR reaction. As amplification proceeds, the nuclease activity of Taq polymerase cleaves the reporter dye from the perfectly hybridized probe, resulting in increased fluorescence. Only the perfectly hybridized probe (wild-type or alternative) were cleaved by the Taq polymerase, resulting in the real-time detection of the PCR products. The ratio of the fluorescence from the two reporter dyes after the PCR reaction determined the genotype of the sample.

After the extracted DNA samples were normalized by diluting them into equal concentrations, qPCR was performed using TaqMan PCR mixture Universal MasterMix II with Uracil-N-glycosylase (UNG) (Applied Biosystems (AB) part of TFS, Foster, CA, USA), TaqMan SNP Genotyping Assay (AB, TFS), and QuantStudio 5 Real-Time PCR System (AB, TFS) according to the manufacturer’s recommendations. PCR thermal conditions were adjusted according to the used TaqMan PCR mixture and were as follows: initial denaturing at 95 °C for 10 min, 40 cycles of 95 °C for 15 sec (denaturing), and 60 °C for 1 min (annealing/extension).To assess the test validity, three positive controls per assay were used: samples that represent three genotype classes (homozygous major, minor, and heterozygous). Genotyping data was visualized using QuantStudio 5 Real-Time PCR System program (version 1.3, AB, TFS).

### 2.5. Statistical Analysis

Statistical analysis for the demographic, clinical, and biochemical characteristics first was performed using descriptive statistics. Frequency distribution for categorical variables was determined by the Chi-square test. Parametric (*t*-test, ANOVA) tests for normally distributed variables and non-parametric (Wilcoxon, Kruskal–Wallis) tests for not normally distributed data were applicable for the subjects clinical and biochemical parameters and the association with genotype/allele frequency after the Shapiro–Wilk normality test was applied. A follow-up, post-hoc test (Dunn’s multiple comparison test) was performed to determine which groups were different from the others when the non-parametric ANOVA was significant. For the association of two normally or not normally distributed variables, the Pearson or Spearman rank correlation coefficients were calculated, respectively. RA risk and genotype/allele association strength was assessed by computing the odds ratio (OR). To distinguish healthy controls from RA patients, logistic regression and ROC curve analysis was applied. Statistically significant results were set at *p* < 0.05. Statistical analysis and data visualization were performed using Microsoft Office Excel 2016 (Microsoft Corporation, Redmond, WA, USA), R (Version 1.1.383, RStudio Inc, Boston, MA, USA), GraphPad Prism (version 7.03, GraphPad Software, San Diego, CA, USA), MedCalc (Version 14.8.1, MedCalc Software Ltd., Ostend, Belgium), and Stata/MP 13.0 (StataCorp LP, College Station, TX, USA) programs.

## 3. Results

### 3.1. Vitamin D Level and Its Correlation with Disease Activity Parameters

In total, 386 subjects participated in the study from January 2017 until December 2019 with seasonal intervals, as recorded before. Altogether, researchers enrolled 206 RA patients, comprising 184 females and 22 males, of which 76 participants were using vitamin D supplements and 130 were not, 109 were treated with biological disease-modifying anti-rheumatic drugs (bDMARDs), and 97 were treated with conventional synthetic DMARDs (e.g., Methotrexate, Sulfasalazine, Hydroxychloroquine, etc.). The mean age of the RA subjects at enrolment was 55.01 ± 11.08 years, andthe mean disease duration was 11.71 ± 9.22 years. For comparison analysis, 180 age- and sex-matched healthy controls, comprising 163 females and 17 males with a mean age of 53.15 ± 10.68, were enrolled. The fully descriptive subject data analysis is presented in Table 1.

**Figure 1:**
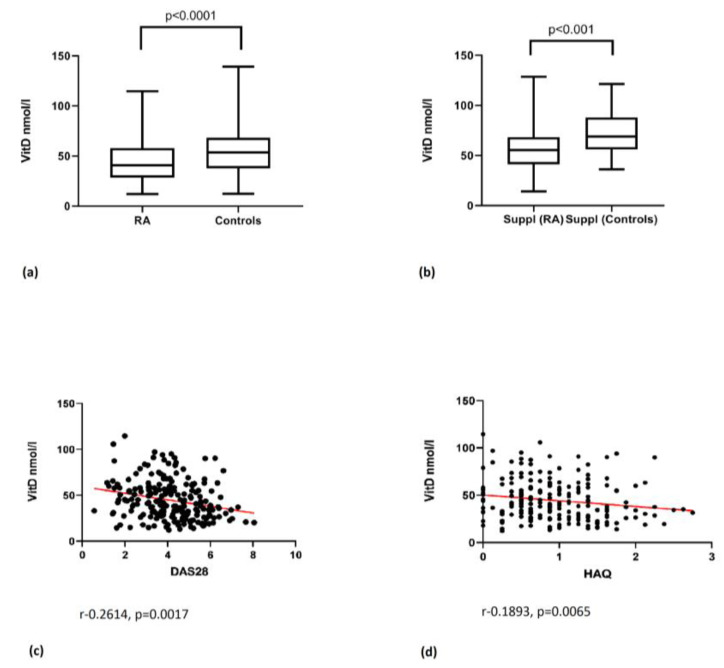
Vitamin D level and correlation with clinical parameters of RA. (**a**) Vitamin D (vitD) level in rheumatoid arthritis (RA) and controls; (**b**) vitamin D level in vitamin D supplemented (Suppl) RA and controls; (**c**) vitamin D correlation with disease activity score 28 C-reactive protein (DAS28 CRP); (**d**) vitamin D correlation with health assessment questionnaire (HAQ).

We registered 25(OH)D deficiency (<50 nmol/L) in 61.55% of RA patients (*n* = 127), insufficiency (≥50–75 nmol/L) in 27.67% of RA patients (*n* = 57), and normal concentrations (≥75–125 nmol/L) in only 9.7% (*n* = 20) of RA patients. The mean serum concentration of 25(OH)D in RA patients was 44.96 ± 21.92 (nmol/L), being significantly lower than in the healthy controls, who had a mean concentration of 54.90 ± 22.82 (nmol/L), with *p* < 0.0001 (Figure 1a). Groups subdivided by vitamin D supplementation also revealed significant differences between the RA patients and controls, with *p* < 0.001 (Figure 1b). Our data confirmed a statistically significant inverse correlation between the vitamin D level, DAS28 CRP, and HAQ scores in RA patients, respectively (r-0.2614, *p* = 0.0017; r-0.1893, *p* = 0.0065) (Figure 1c,d).

On the contrary, vitamin D levels had no significant correlation with the RAID score. However, patients using vitamin D supplementation had higher vitamin D levels and a lower RA DAS28 CRP score (*p* < 0.05), suggesting that higher vitamin D concentration is associated with lower disease activity.

### 3.2. VDR Polymorphism Analysis

Genotype frequencies of RA patients and healthy controls were tested for compatibility with the Hardy–Weinberg equilibrium, confirming *FokI*, *ApaI*, and *BsmI VDR* loci compatibility, except for *BsmI* in RA subjects (*p* = 0.045). No significant association between the overall risk of RA disease for any allele or genotype of the four *VDR* loci tested was found (Table 2). There was also no significant difference between RA patients and healthy controls regarding the distribution of *BsmI*, *ApaI*, *FokI*, and *TaqI* genotypes and their allele frequencies (*p* > 0.05).

Vitamin D levels comparing RA patients and healthy controls in reference to four *VDR* polymorphisms were significantly different in both groups (*p* < 0.005), except for the *TaqI tt*, *BsmI bb*, and *FokI ff* genotypes (Table 3). Vitamin D concentration in RA patients and controls carrying minor alleles of *TaqI*, *BsmI*, and *FokI* variants did not differ significantly and showed similar concentrations. However, our data showed a strong tendency that the *TaqI tt* genotype had higher vitamin D levels vs. the *Tt* genotype as well as the *BsmI bb* vs. *Bb* genotype in RA patients (*p* = 0.0597, *p* = 0.1145 respectively).

**Figure 2:**
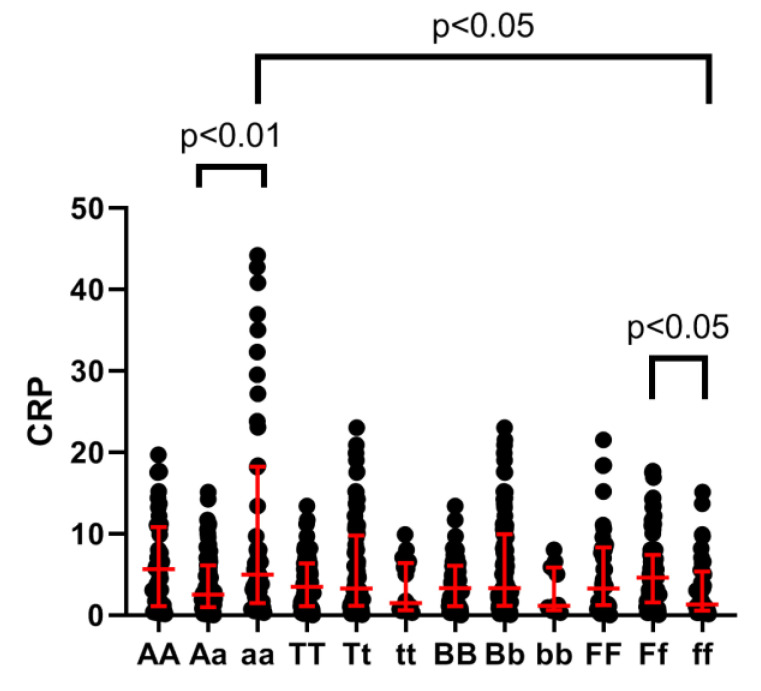
C–reactive protein (CRP) level and vitamin D receptor gene polymorphisms in rheumatoid arthritis patients.

The CRP level (mg/l) in disease-affected subjects was significantly higher in the *ApaI aa* genotype vs. *Aa* (*p* = 0.0049), in the *FokI Ff* genotype vs. *ff* (*p* = 0.0162), and in the *aa* vs. *ff* (*p* = 0.0061), suggesting that the *ApaI aa* variant is associated with a higher CRP concentration in RA subjects (Figure 2). However, there was no significant association between the DAS28 CRP, HAQ, or RAID score and any *VDR* gene polymorphism variant.

**Figure 3:**
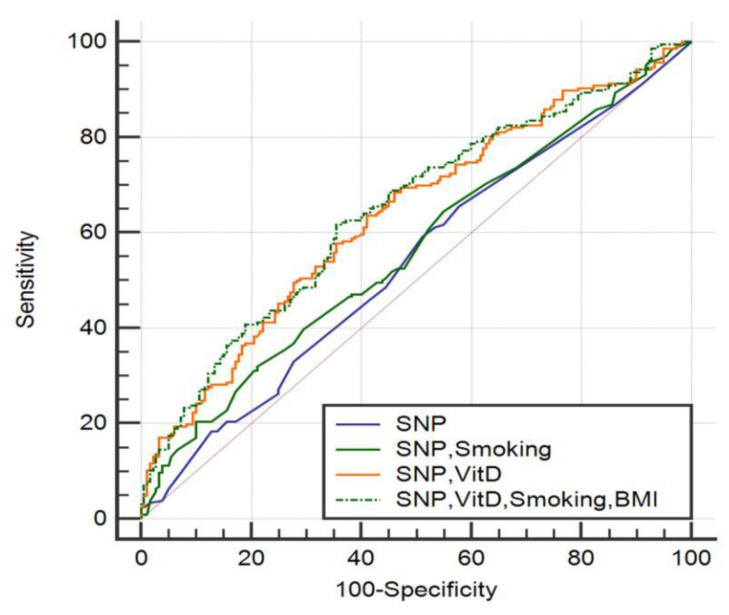
ROC curve adjusted for the single nucleotide polymorphisms (SNP), vitamin D, body mass index (BMI), and smoking models.

A logistic regression analysis adjusted for SNP, vitamin D, body mass index (BMI), and smoking revealed that the combination of all the listed variable models best distinguishes RA-affected subjects from healthy controls compared to the SNP model alone (95% CI, confidence interval (0.597, 0.695), *p* = 0.0088), with a sensitivity of 71.84%, and a specificity of 50.00%. The ROC curve graphic rendering is shown in Figure 3.

In the single nucleotide polymorphism (SNP) model, the area under the (ROC) curve (AUC) was 0.537 (95% CI (0.486, 0.588)), and *p* > 0.05; in the SNP, smoking model, the AUC was 0.564 (95% CI (0.513, 0.614)), and *p* > 0.05; in the SNP, vitamin D (VitD) model the AUC was 0.637 (95% CI (0.587, 0.685)), and *p* = 0.0146; in the SNP, VitD, smoking, and body mass index (BMI) model the AUC was 0.647 (95% CI (0.597, 0.695), and *p* = 0.0088.

## 4. Discussion

Vitamin D’s role in RA etiopathogenesis and its relation with the disease’s clinical course has been studied in experimental, case-control, and clinical vitamin D supplementary studies over the past decades. The data are controversial and differ according to geographical latitude, ethnicity, environmental factors, study design, etc. However, conducting numerous in vitro and in vivo studies, it was proved that immune cells prone in RA pathogenesis are sensitive to active vitamin D form 1,25(OH)2D_3_, and by downregulating microRNA-22 expression, it induces anti-inflammatory responses foreseen as reduced fibroblast-like synoviocytes proliferation, osteoclast-mediated bone, cartilage destruction, and lower levels of pro-inflammatory cytokines [22,23]. Furthermore, VDR signaling-deficient transgenic mice with experimental chronic arthritis showed increased progression of erosive arthritis [24]. The present study analyzed the prevalence of vitamin D deficiency among RA patients and its possible association with RA disease activity and disability scores, as well as the role of *VDR* polymorphisms and risk of RA.

The study revealed a high incidence of vitamin D deficiency in RA patients despite the supplementary use of vitamin D, occurring in 61.55% of patients.Vitamin D deficiency is common among RA patients and may play a complementary role in increased RA risk, disease activity, and quality of life. A meta-analysis of prospective studies of 215,757 subjects and 874 incident cases of RA suggested that a greater use of vitamin D supplements reduced RA development risk by 24.2% [25]. The results of an observational, cross-sectional Comorbidities in Rheumatoid Arthritis (COMORA) study performed in 15 countries worldwide showed that the highest vitamin D insufficiency levels among RA patients were foundin Korea (71%), whereas the lowest were found in Italy (36%) [26]. Similar results were reported in a European multicenter pilot survey conducted in 13 European countries, which involved 625 RA patients and 276 age- and sex-matched healthy controls. Vitamin D deficiency (<20 ng/mL) was observed in 66% of RA patients, also showing differences between several countries, where the Spanish population presented higher vitamin D levels compared to Lithuanian, Latvian, and Polish populations, among others [27].

Similar to our reported results, significant differences in the vitamin D level between RA and control groups, and an inverse correlation between vitamin D levels, RA disease activity (DAS 28), and disability measurements (HAQ) have been reported in numerous studies and meta-analyses to support the hypothesis [25,26,27,28,29,30]. Additionally, an exploratory study investigating the role of vitamin D supplementation in RA disease activity course contributed to the significant improvement in DAS28 CRP score in vitamin D deficient patients within 3 months of treatment [31]. Nevertheless, data from similar design studies arecontradictory. As tudy of Polish population with a small sample size showed no differences between RA patients and healthy controls regarding vitamin D level nor a correlation with the DAS 28 score [32]. The same results were published by Higgins et al. [33] and showed that lower levels of vitamin D correlated with a higher range of visual analogue scale (VAS), but not with the DAS 28 tool.

The present case–control study demonstrated no significant associations between *VDR* polymorphisms and increased RA disease risk, andno allele or genotype frequency differences were observed in RA patients and controls. Similar to our results, a small Italian and German study and a study of 120 RA patients and 200 healthy individuals of the Spanish population showed no differences in *VDR* polymorphisms frequencies and RA predisposition [34,35,36]. Only the *TaqI tt* genotype, compared to *TT* and *Tt*, showed earlier disease onset [34]. Numerous other published studies have shown abroad spectrum of results that were summarized in few meta-analyses [14,37,38]. Pooled data from seven studies showed no association between RA and *VDR* polymorphisms in the entire cohort of studied subjects (OR = 1.1740, 95%, CI = 0.994–1.387, *p* = 0.059). However, groups subdivided by ethnical origin revealed a significant association between the *F* allele and RA in Europeans (OR = 1.402, 95% CI = 1.126–1.746, *p* = 0.003) [14]. The same results were also published in another meta-analysis of four Asian and five European studies, as well as in a French RA population analysis [37,38]. The focus on genetic variants of the *VDR* gene and possible susceptibility to RA was raised because of the partial non-human leukocyte antigen (HLA) genes disease etiology and its involvement in vitamin D functioning mediated through VDR located on monocytes, activated lymphocytes, and other cells important in joint inflammation genesis. The importance of the *VDR* gene is also presented in an in vivo mouse model study where peripheral T lymphocytes with inactive VDRs demonstrated indifference on vitamin D effect in autoimmune disease development [39].

Additionally, our study results showed a tendency of higher vitamin D levels in the *TaqI tt* and *BsmI bb* RA groups, and vitamin D levels in healthy controls and RA patients carrying the same *FokI ff*, *TaqI tt*, and *BsmI bb* genotype did not differ significantly. This insight suggests a possible role of certain genotypes in vitamin D metabolism. However, there was no significant association between RA activity or disability scores with any *VDR* gene variant. Contrary to our results, in a recently published article on *VDR* [40], two vitamin D bioactivating cytochrom P450 isoforms (*CYP27A1*, *CYP2R1*), gene polymorphisms, and lipid profile in RA showed that vitamin D deficiency was an independent predictor of higher DAS 28, HAQ scores, and affected joint counts exclusively in the *VDR FokI Ff/ff* genotype group in a multivariate regression analysis (*p* < 0.05). Though the vitamin D level itself was not associated with disease activity or quality of life in the general cohort [41]. It is known that *FokI* and *BsmI* are functioning sites of the *VDR* gene; moreover, *FokI* determines the production of the different length protein. The short *F* allele protein is more active and possibly leads to lower vitamin D levels in the *FF* genotype compared to *ff* [40]. The homozygous short *FF* genotype present in human monocytes and dendritic cells after stimulation with IFN-γ expressed higher levels of IL-12 by means of IL-12p40, IL12p35 mRNA, and IL-12p70 protein compared to the *ff* genotype detected cells. This evidences that *FokI* polymorphism affects immune cells actions and may have an effect in autoimmune disease pathogenesis [42].

Despite the strength in the homogeneity of the RA and control groups and demonstration of significant associations, the present study has some weaknesses. Statistical insignificance of the *VDR* gene polymorphisms analysis could be explained by an insufficient study cohort due to the rather small Lithuanian population, and thus, needs to be replicated in larger scale studies. However, this could also prove ethnic differences or the questionable effect of *VDR* gene in RA etiopathogenesis. Another limitation is that the study design demonstrated a possible link between RA and vitamin D deficiency, but did not necessarily revealed the cause-and-effect fact because RA is a multifactorial etiology disease. This also suggests that further investigations are obligatory.

## 5. Conclusions

In conclusion, the study confirmed that vitamin D deficiency is prevalent among RA patients and that the 25(OH)D level is significantly lower compared with healthy controls. Lower vitamin D concentrations are associated with increased disease activity scores, higher disability rates, and therefore, a worse quality of life, supporting a possible therapeutic role of vitamin D deficiency treatment in RA patients. However, the genetic analysis of four *VDR* polymorphisms did not confer the susceptibility to RA in the Lithuanian population. Notwithstanding, to our knowledge, it is the first *VDR* polymorphism study of RA in the Lithuanian population that suggests some of gene variants that may be taking part in multifactorial vitamin D metabolism, and possibly in RA clinical course.

## Figures and Tables

**Table 1:** Demographical, clinical, and biochemical characteristics of rheumatoid arthritis (RA) patients and controls.

Parameters	RA (*n* = 206)	Controls (*n* = 180)	*p* Significance
Gender (Male/Female)	22/184	17/163	0.6879
Age	55.01 ± 11.08	53.15 ± 10.68	0.0975
BMI (kg/m^2^)	25.62 ± 4.64	26.46 ± 4.74	0.1005
25(OH)D (nmol/L):	44.96 ± 21.92	54.90 ± 22.82	*p* < 0.0001 *
Deficiency	127 (61.65)	79 (43.89)	
Insufficiency	57 (27.67)	73 (40.56)	
Normal range	20 (9.7)	28 (15.56)	
Smoking (n):			0.0619
Yes	34	18	
No	172	162	
Disease onset (Years)	43.30 ± 12.80		-
RA duration (Years)	11.71 ± 9.22		-
DAS28 CRP:	4.16 ± 1.46		-
High activity	56 (27.18)		
Moderate activity	98 (47.57)		
Low activity	19 (9.22)		
Remission	33 (16.01)		
HAQ	0.95 ± 0.61		-
RAID	4.74 ± 2.23		-
CRP (mg/L)	12.55 ± 21.7		-
VAS (mm)	45.57 ± 20.88		-

Values represent the mean ± standard deviation or *n*,%; *, Statistically significant values. RA, rheumatoid arthritis; BMI, body mass index; DAS 28, disease activity score 28; CRP, C-reactive protein, HAQ, health assessment questionnaire; RAID, rheumatoid arthritis impact of disease score; VAS, visual analogue scale.

**Table 2:** Vitamin D receptor (*VDR*) gene genotype and allele frequency distribution in RA and controls.

	Genotype Frequency *n* (%)				Allele Frequency *n* (%)			OR (95% CI)	HWE
*ApaI*	*AA*	*Aa*	*aa*	X2(*p*)	*A*	*a*	X2(*p*)		X2(*p*)
RA (*n* = 206)	56 (27)	99 (48)	51 (25)	1.172	211 (51)	201 (49)	0.322	0.983 (0.618–1.564)	0.302 (0.583)
Controls (*n* = 180)	41 (23)	95 (53)	44 (24)	(0.557)	177 (49)	183 (51)	(0.570)	1.085 (0.818– 1.440)	0.561 (0.454)
*TaqI*	*TT*	*Tt*	*tt*		*T*	*t*			
RA (206)	95 (46)	92 (45)	19 (9)	0.014	282 (68)	130 (32)	0.002	1.041 (0.519–2.092)	0.237 (0.626)
Controls (180)	83 (46)	81 (45)	16 (9)	(0.993)	247 (69)	113 (31)	(0.961)	1.008 (0.743–1.366)	0.360 (0.548)
*BsmI*	*BB*	*Bb*	*bb*		*B*	*b*			
RA (206)	91 (44)	101 (49)	14 (7)	0.036	283 (69)	129 (31)	0.001	0.937 (0.428– 2.049)	4.028 (0.045)
Controls (180)	80 (45)	87 (48)	13 (7)	(0.982)	247 (69)	113 (31)	(0.981)	0.996 (0.735– 1.351)	2.685 (0.101)
*FokI*	*FF*	*Ff*	*ff*		*F*	*f*			
RA (206)	43 (21)	110 (53)	53 (26)	0.085	196 (48)	216 (52)	0.045	1.071 (0.675– 1.699)	1.023 (0.312)
Controls (180)	38 (21)	98 (55)	44 (24)	(0.958)	174 (48)	186 (52)	(0.832)	1.031 (0.777– 1.368)	1.461 (0.227)

VDR, vitamin D receptor; RA, rheumatoid arthritis; OR, odds ratio; 95% CI, Confidence interval; HWE, Hardy–Weinberg equilibrium; X2, Chi-square; *p*, *p* value.

**Table 3:** Mean vitamin D level in RA and controls grouped by the *VDR* polymorphism genotypes.

Genotype	RA	Controls	*p* Value
	Vitamin D Level (nmol/L, M ± SD)		
*ApaI*			
AA	43.46 ± 23.52	54.89 ± 21.7	0.0034 *
Aa	45.3 ±22.37	53.71 ± 23.05	0.009 *
aa	45.87 ± 19.44	57.49 ± 23.64	0.0087 *
*TaqI*			
TT	46.2 ± 20.65	56.47 ± 24.3	0.0014 *
Tt	42.86 ± 24.29	53.11 ± 21.33	0.0003 *
tt	48.88 ± 14.8	55.86 ± 23.02	0.2869
*BsmI*			
BB	46.62 ± 20.71	56.78 ± 23.66	0.0012 *
Bb	43.15 ± 23.97	53.62 ± 22.16	0.0002 *
bb	47.16 ± 11.9	51.89 ± 22.83	0.8952
*FokI*			
FF	42.54 ± 18.62	54.99 ± 24.1	0.0123 *
Ff	44.97 ± 23.75	56.12 ± 22.08	<0.0001 *
ff	43.4 ± 20.59	46.0 ± 23.62	0.2143

RA, rheumatoid arthritis; VDR, vitamin D receptor; M ± SD, mean ± standard deviation; *, Statistically significant values.

## Data Availability

The original datasets are not publicly available due to data protection policies. The data presented in this study are available on scientific request from the corresponding author.
